# CT-based radiomics predicts HRD score and HRD status in patients with ovarian cancer

**DOI:** 10.3389/fonc.2024.1477759

**Published:** 2025-01-08

**Authors:** Yujiao Wu, Qianhui Zhang, Wenyan Jiang, Yuhua Gao, Bin Qu, Xingling Wang

**Affiliations:** ^1^ School of Intelligent Medicine, China Medical University, Liaoning, China; ^2^ Department of Scientific Research and Academic, Cancer Hospital of China Medical University, Liaoning Cancer Hospital and Institute, Shenyang, China; ^3^ Department of Gynecology, Liaoning Cancer Hospital and Institute, Cancer Hospital of China Medical University, Shenyang, China

**Keywords:** ovarian cancer, HRD score, HRD status, CT, radiomics

## Abstract

**Introduction:**

This study predicted HRD score and status based on intra- and peritumoral radiomics in patients with ovarian cancer (OC) for better guiding the use of PARPi in clinical.

**Methods:**

A total of 106 and 95 patients with OC were included between January 2022 and November 2023 for predicting HRD score and status, respectively. Radiomics features were extracted and quantitatively analyzed from intra- and peri-tumor regions in the CT image. Radiomics signatures (RSs) were built based on features from intra- and peri-tumor regions for predicting HRD score and status alone or in combination. Subject working characteristics (ROC) area under the curve (AUC), sensitivity (SEN), and specificity (SPE) were calculated as comparative metrics.

**Results:**

For predicting HRD score, 4 and 2 features were selected as the most important predictors from the intra- and peritumoral regions, respectively. For predicting HRD status, 4 features from the intratumoral region and 2 from the peritumoral region were identified as the most important predictors. Radiomics nomograms created by combining RSs and important clinical factors showed good predictive results with AUCs of 0.852 (95% confidence interval [CI]: 0.765-0.938, SEN = 0.907, SPE = 0.655) and 0.781 (95% CI: 0.621-0.941, SEN = 0.688, SPE = 0.833) in the training and validation cohort for predicting HRD score, respectively; with AUCs of 0.874 (95% CI: 0.790-0.957, SEN = 0.765, SPE = 0.867) and 0.824 (95% CI: 0.663-0.985, SEN = 0.762, SPE = 0.800) in the training and validation cohort for predicting HRD status, respectively.

**Discussion:**

Calibration curves and decision curve analysis (DCA) confirmed potential clinical usefulness of our nomograms. Our findings suggest that radiomics features derived from the CT image of OC have the potential to predict HRD score and status, and the developed nomograms can enrich the range of applicable population of PARPi, prolong progression-free survival and provide personalized treatment for OC patients.

## 1 Introduction

Ovarian cancer (OC) is the most common cancer among women and the leading cause of death from gynecologic cancers worldwide (1). OC is classified into epithelial ovarian cancer (EOC, accounting for approximately 90%) and non-epithelial ovarian cancer (NEOC, accounting for about 10%), which differ in molecular characteristics, treatment outcomes, and prognosis. (2). Standard therapy for OC includes primary surgical cytoreduction and platinum-based chemotherapy (3). However, the PI3K pathway plays a crucial role in chemotherapy resistance and genomic stability in EOC, being frequently upregulated in many cancers, including OC, and involved in key processes such as DNA replication and cell cycle regulation, which activation enhances cell survival and repair mechanisms, contributing to chemotherapy resistance. (4). According to previous studies (5), 70% of tumors will recur within 6 months after the last dose of the platinum-based chemotherapy due to platinum resistance in the tumor (6). Proteomic s technologies such as mass spectrometry and protein arrays are key to understanding platinum drug resistance. They identify the molecular signatures and proteomic features that drive resistance, identify new therapeutic targets and lay the foundation for the development of targeted therapies (7). Recently, targeted therapies involving the poly (ADP-ribose) polymerase inhibitor (PARPi) and/or the anti-angiogenic agent bevacizumab (8, 9) have become the new standard for treating platinum-sensitive OC patients, and the use of PARPi has been found to be effective in prolonging survival in patients with platinum-sensitive and recurrent OC (10).

There have been six primary pathways of DNA damage response (DDR) identified, which are variably used to address double-strand DNA breaks (DSB) and single-strand DNA breaks (SSB) damage from a variety of mechanisms of injury. Homologous recombination repair (HRR) and nonhomologous end joining (NHEJ) recombination are the two major pathways responsible for repairing DSB (11), however, The Cancer Genome Atlas (TCGA) suggests that Homologous Recombination Deficiency (HRD) is present in approximately half of OC patients (12). The majority of HRD tumors will occur in OC patients with germline breast cancer susceptibility gene1/2 (BRCA1 and BRCA2) mutations, however, there are also patients with germline mutations in other homologous recombination pathway genes (13). OC patients with HRD-positive usually exhibit distinct clinical phenotypes, which include superior response to platinum-based chemotherapy and sensitivity to PARPi (14). Therefore, HRD testing is clinically significant in guiding the use of PARPi and the developing subsequent treatment plans.

In the clinical setting, tumors can be analyzed using the MyChoice HRD Plus assay (Myriad Genetic Laboratories Inc, Salt Lake City, UT), and HRD-positive has been defined as genomic instability score (GIS) ≥ 42 (high HRD score). HRD-negative was defined as GIS < 42 (low HRD score) and no tumor mutations in BRCA1/2 (15). However, the HRD testing has not yet been integrated into routine clinical practice due to the high cost (16). In contrast to HRD testing, clinical imaging is noninvasive, low-cost and can reflect a wide range of tumoral heterogeneity. Despite contrast-enhanced computed tomography (CT) is the imaging modality of choice for staging and treatment follow-up of OC (17), radiologists cannot predict patients’ HRD status based on visual assessments of medical images due to the lack of imaging markers.

Radiomics refers to noninvasive quantification of tumor characteristics from images, and has been suggested as a promising and challenging field in recent years (18). Numeric radiomics features can be calculated and selected from imaging data to quantify and to screen tumor phenotypes, followed by construction of machine learning-based models to aid in diagnosis, prognosis and prediction of treatment responses in oncology (19, 20). Recent radiomics studies on OC have highlighted that medical images contain a wealth of potential information regarding prognosis (21, 22). and genetic status (23, 24). A recent study demonstrated significant associations between radiomics features derived from the CT image of OC and the risk of disease progression (25). In another study for predicting the genetic status in OC, zhang et al. built a nomogram based on CT-based radiomics features and clinical characteristics, and showed that the nomogram had good predictive performance in predicting BRCA mutations (26).

Predicting only BRCA mutations ignores HRD in tumors caused by other reasons (23, 24, 26), and predicting HRD status may enrich the applicable population of PARPi. HRD is relatively increased prevalence in OC compared to pancreatic, liver, lung and kidney cancers (13), and to the best of our knowledge, there is no radiomics study predicting the HRD score or status of patients with OC (27). Pathological studies have shown that not only the internal regions of ovarian tumors but also the normal tissues and stroma surrounding the tumor are enriched with a variety of potential markers associated with cancer invasion and metastasis. These markers may provide valuable information for predicting HRD, which warrants further investigation. (28). Therefore, this study explored values of radiomics features of Intra− and peritumoral regions in patients with OC to predict HRD score and HRD status, aiming to enrich the range of applicable population of PARPi, prolong progression-free survival and provide personalized treatment for OC patients.

## 2 Methods

### 2.1 Patients

**Figure 1:**
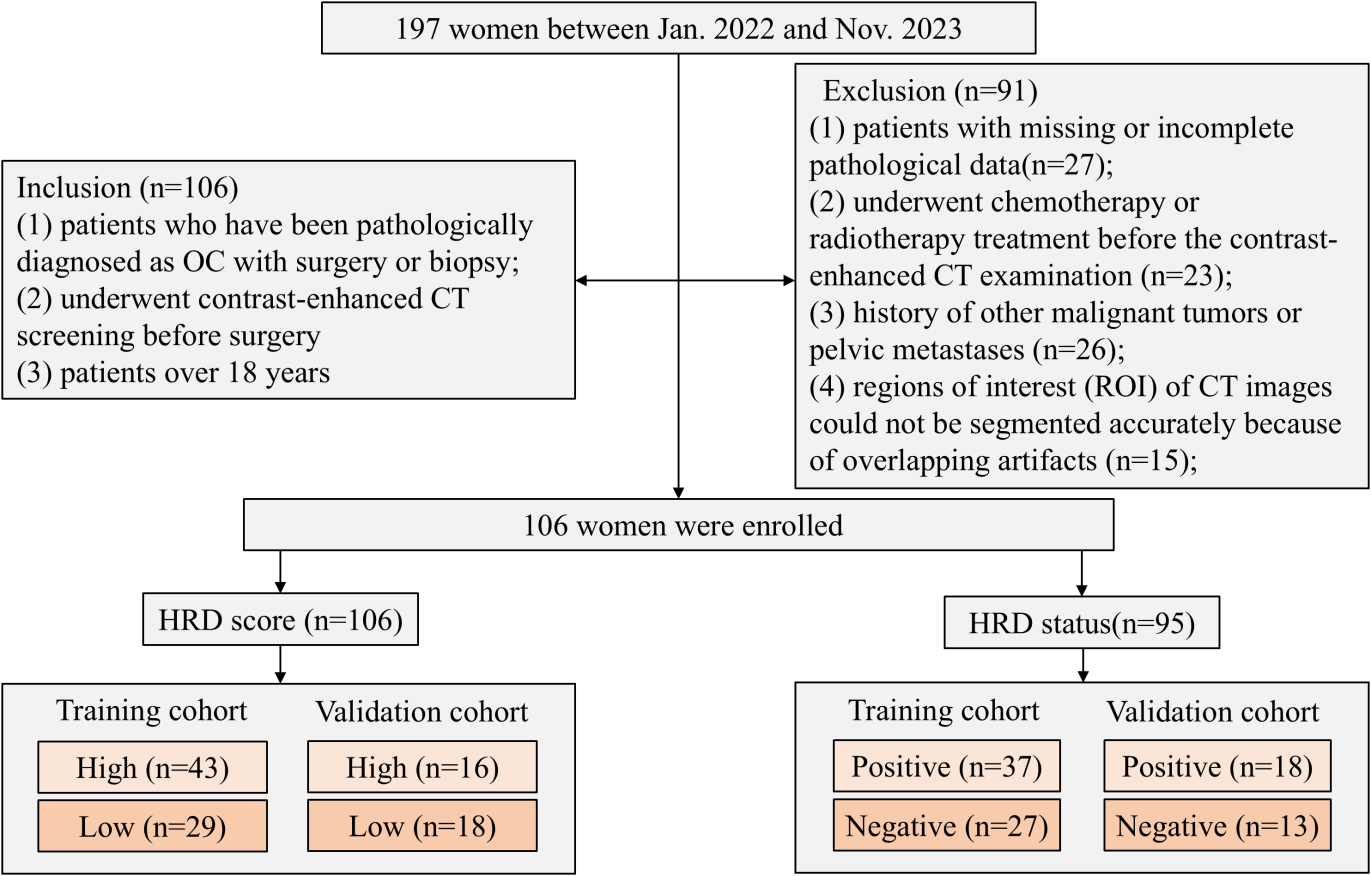
Details of the process of inclusion and exclusion of patients.

The retrospective study was approved by our institutional review board (20220788). A cohort of 197 female patients was constructed between Jan. 2022 and Nov. 2023. Inclusion criteria were as follows: (1) patients who had pathologically diagnosed as OC with surgery or biopsy; (2) underwent contrast-enhanced CT screening before surgery; and (3) patients over 18 years. Exclusion criteria were as follows: (1) patients with missing or incomplete pathological data; (2) underwent chemotherapy or radiotherapy treatment before the contrast-enhanced CT examination; (3) a history of other malignant tumors or pelvic metastases; and (4) regions of interest (ROI) of CT images that could not be segmented accurately because of overlapping artifacts. **Figure 1** shows details of the process of inclusion and exclusion of patients. A total of 106 and 95 patients were finally included for predicting the HRD score and status, respectively. Eleven patients were excluded due to an HRD score below 42 and the absence of BRCA gene testing, resulting in a final cohort of 95 patients with determined HRD status.

Patients were randomly divided into a training and validation cohort at a ratio of 2:1 by stratified sampling. Clinical characteristics contain age, body mass index (BMI), family history, BRCA1/2 mutation status, Federation of International of Gynecologists and Obstetricians (FIGO) and tumor diameter, and were collected from the electronic medical recodes system (EMRS). Pathological data includes histotype, carbohydrate antigen 125 (CA125), proliferation marker protein Ki-67 (Ki-67), estrogen receptor (ER), progesterone receptor (PR) and P53, and were recorded from pathology reports. **Figure 1** shows the inclusion and exclusion flow chart.

### 2.2 CT data acquisition and tumor segmentation

All patients enrolled in this study underwent preoperative contrast-enhanced CT scans using a multi-slice CT scanner (Philips iCT 256) to obtain pelvic CT images. The parameters of CT scan were: 120 kVp and maximum tube current 500 mA. The acquired CT data were stored in an image archiving and communication system. For each patient, a radiologist with 15 years of experience used the software ITK-SNAP (www.ITK-SNAP.org) to draw the tumor region (ROI) that covers the entire tumor region layer by layer. To ensure the ROIs were segmented correctly, a radiologist with 19 years of experience verified all manual depicted ROIs.

Considering that the surrounding area of the tumor may provide predictive information, radial expansion along the boundary of the segmented ROI at a distance of 1cm yields a ring-shaped area, which was achieved using the “SimpleITK” package in Python v.3.6. Thus, the generated new mask represents the peritumoral region. Both the masks of tumor and the progressively expanded peritumoral regions were used for further radiomics analysis.

### 2.3 Radiomics feature extraction

In this study, three categories of radiomics features were extracted from the intra- and peritumoral ROI in the CT image, which include 18 first-order, 26 shape-based and 74 texture features. All features were extracted using the “Pyradiomics” package (3.0.1) in Python v.3.6. The first-order and texture features were also extracted from filtered CT image that were filtered with Wavelet, LoG, Square, Square Root, Logarithm, Exponential, Gradient and Local Binary Pattern filters. Detailed information about the extracted radiomics features was described in the Pyradiomics documentation (https://pyradiomics.readthedocs.io/).

### 2.4 Feature screening and model establishment

The features were evaluated using the intra-class correlation coefficients (ICC) (29), with thirty randomly selected patients. Another radiologist was invited to manually segment the ROIs on each CT slice. Features with ICCs > 0.85 were considered to be reliable and retained. The Mann-Whitney *U* test was used for the significance test, and the feature with P < 0.05 were considered significant. Finally, the features selected by the above two steps were further put into the least absolute shrinkage and selection operator (LASSO) with 5-fold cross-validation for selecting the parameter lambda using the 1 standard error of the minimum criteria (1-SE criteria).

The LASSO selected features were used to establish the radiomic signatures (RSs) for prediction of HRD score and status with the logistic regression model. Specifically, using the Akaike Information Criterion (AIC) as a stopping rule for the stepwise regression method, the subset of features that are most valuable to the prediction goal are selected from the full model containing all features and used to build the logistic regression classification model. (30). A radiomics-clinical combined nomogram was developed using logistic regression analysis.

### 2.5 Statistical analysis

**Figure 2:**
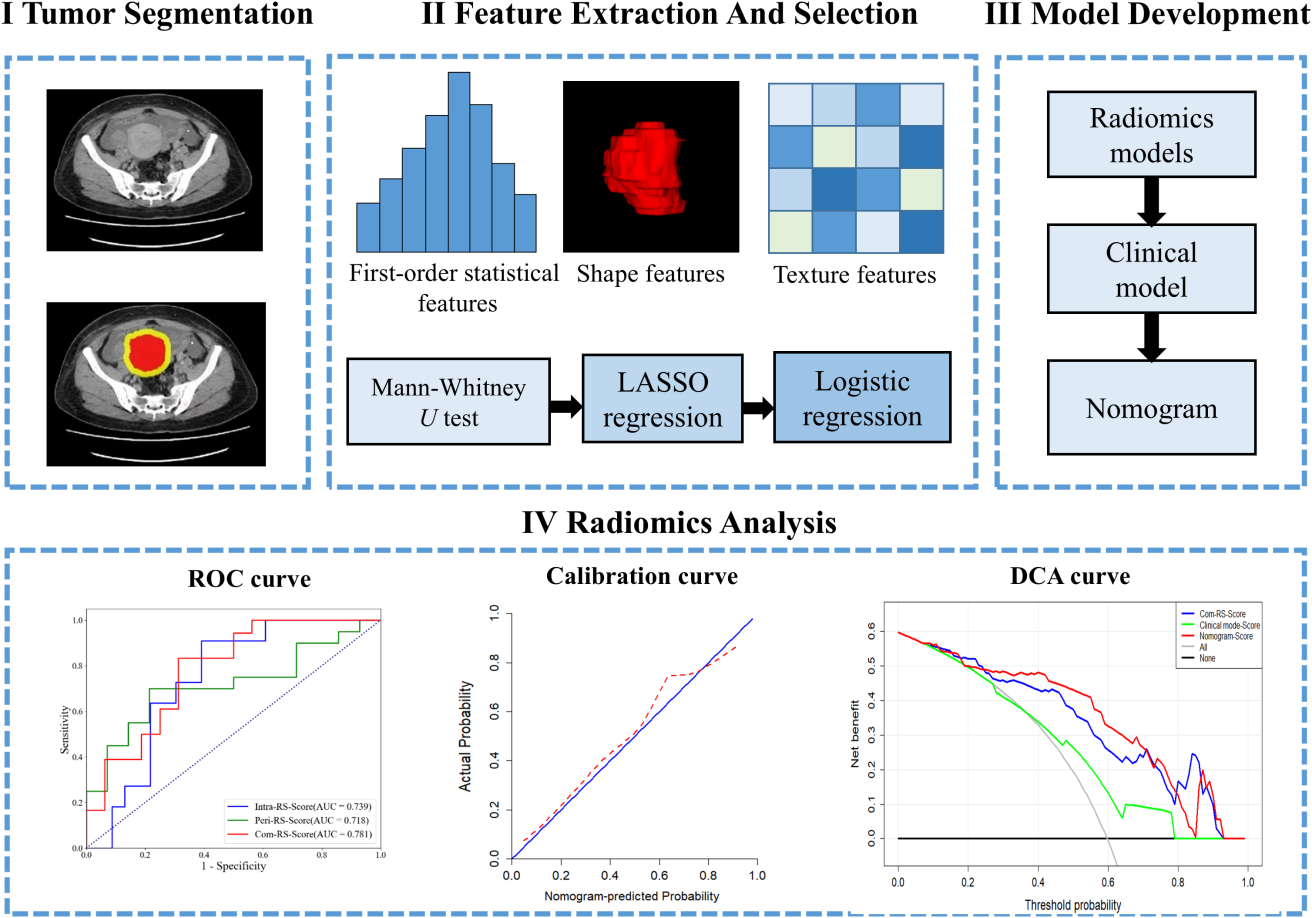
Work flow of the present study.

We use IBM SPSS Statistics 24 based on the type of data for clinical characteristics that the Chisquare (31) and Mann-Whitney *U* tests (32) to assess statistical significances of all clinical factors as appropriate. The statistical significance for all two-sided tests was set at *P* < 0.05. The receiver-operating characteristic (ROC) analysis was performed to compute the area under the ROC curve (AUC) for comparisons among the developed RSs. Delong test (33) is used to compare differences between the models. Performance of the nomogram was evaluated by calibration curve (34) and decision curve analysis (DCA) (35). Statistical analyses were performed in R (version 4.0; R Core Team, Vienna, Austria, https://www.R-project.org). **Figure 2** shows work flow chart of the study.

## 3 Results

### 3.1 Patients’ characteristics

**Table 1** showed characteristics of the patients. For prediction of HRD score, a total of 72 patients were finally included to form the training cohort, which contains 43 high HRD score, 29 low HRD score, and a total of 34 patients were included in the validation cohort including 16 high HRD score, 18 low HRD score. Age, Family history, FIGO Stage, Serous Carcinoma and Ki-67 were considered statistically significant different (*P*<0.05) in training cohort, but just family history and Ki-67 were considered statistically significant different (*P*<0.05) in both training and validation cohorts.

**Table 1:** Clinical and pathologic characteristics of the patients.

Characteristic	Training (n=72)		*P*	Validation (n=34)		*P*	Training (n=64)		*P*	Validation (n=31)		*P*
	High HRD score (n=43)	Low HRD score (n=29)		High HRD score (n=16)	Low HRD score (n=18)		HRD-positive (n=37)	HRD-negative (n=27)		HRD-positive (n=18)	HRD-negative (n=13)	
Age(mean ± SD)	53.05 ± 8.21	58.66 ± 8.55	0.009^*^	58.75 ± 9.59	59.78 ± 10.16	0.597	54.08 ± 8.94	59.26 ± 8.63	0.030^*^	55.67 ± 9.82	60.77 ± 9.83	0.196
BMI(median(range))	22.95 (17.95, 35.65)	22.49 (18.67, 28.98)	0.585	21.29 (16.38, 28.25)	23.97 (17.68, 27.05)	0.266	22.02 (17.96, 29.55)	23.28 (17.68, 28.98)	0.268	21.59 (16.38, 35.65)	22.03 (18.67, 27.05)	0.650
Family history, n. (%)												
Yes	17 (23.61)	4 (5.56)	0.018^*^	7 (20.58)	4 (11.78)	0.042^*^	12 (18.75)	3 (4.69)	0.047^*^	9 (29.03)	2 (6.45)	0.047^*^
No	26 (36.11)	25 (34.72)		9 (26.47)	14 (41.17)		25 (39.06)	24 (37.50)		9 (29.03)	11 (35.48)	
FIGO Stage, n. (%)												
Stage I-II	5 (6.94)	13 (18.06)	0.001^*^	3 (8.82)	0 (8.82)	0.054	6 (9.38)	2 (3.13)	0.293	1 (3.23)	1 (3.23)	0.811
Stage III-IV	38 (52.78)	16 (22.22)		13 (38.24)	18 (44.12)		31 (48.44)	25 (39.06)		17 (54.84)	12 (38.91)	
Serous Carcinoma												
Yes	42 (58.33)	24 (33.33)	0.025^*^	15 (44.12)	13 (38.24)	0.100	36 (56.25)	21 (38.81	0.013^*^	15 (48.39)	11 (35.48)	0.924
No	1 (1.39)	5 (6.94)		1 (2.94)	5 (4.71)		1 (1.56)	6 (9.38)		3 (9.68)	2 (6.45)	
Ki-67, n. (%)												
≥50%	40 (55.56)	14 (19.44)	<0.001^*^	14 (41.18)	9 (26.47)	0.020^*^	34 (53.13)	19 (29.69)	0.024^*^	17 (54.84)	8 (25.81)	0.022^*^
<50%	3 (4.17)	15 (20.83)		2 (5.88)	9 (26.47)		3 (4.69)	8 (12.50)		1 (3.23)	5 (16.13)	
ER, n. (%)												
Positive	39 (54.17)	22 (30.56)	0.086	14 (41.18)	16 (47.06)	0.900	33 (51.56)	23 (35.94)	0.632	15 (48.39)	11 (35.48)	0.924
Negative	4 (5.56)	7 (9.72)		2 (5.88)	2 (5.88)		4 (6.25)	4 (6.25)		3 (9.68)	2 (6.45)	
PR, n. (%)												
Positive	31(54.17)	21 (29.17)	0.976	8 (23.53)	12 (35.29)	0.324	27 (42.19)	19 (29.69)	0.819	12 (38.71)	8 (25.81)	0.768
Negative	12 (16.67)	8 (11.11)		8 (23.53)	6 (17.65)		10 (15.63)	8 (12.50)		6 (19.35)	5 (16.13)	
P53, n. (%)												
Mutant-type	38 (52.78)	21 (29.17)	0.084	14 (41.18)	14 (41.18)	0.458	34 (53.13)	20 (31.25)	0.053	14 (45.16)	9 (19.03)	0.592
Wild-type	5 (6.94)	8 (11.11)		2 (5.88)	4 (11.76)		3 (4.69)	7 (10.94)		4 (12.90)	4 (12.90)	

SD, standard deviation; BMI, body mass index; FIGO, Federation of International of Gynecologists and Obstetricians; Ki-67, proliferation marker protein; ER, estrogen.

receptor; PR, progesterone receptor HRD, homologous recombination deficiency *p < 0.05.

For prediction of HRD status, a total of 64 patients were finally included to form the training cohort, which contains 37 HRD-positive, 27 HRD-negative, and a total of 31 patients were included in the validation cohort, including 18 HRD-positive, 13 HRD-negative. Family history and Ki-67 were considered statistically significant differences in both training and validation cohorts.

### 3.2 Radiomics feature selection and analysis

**Figure 3:**
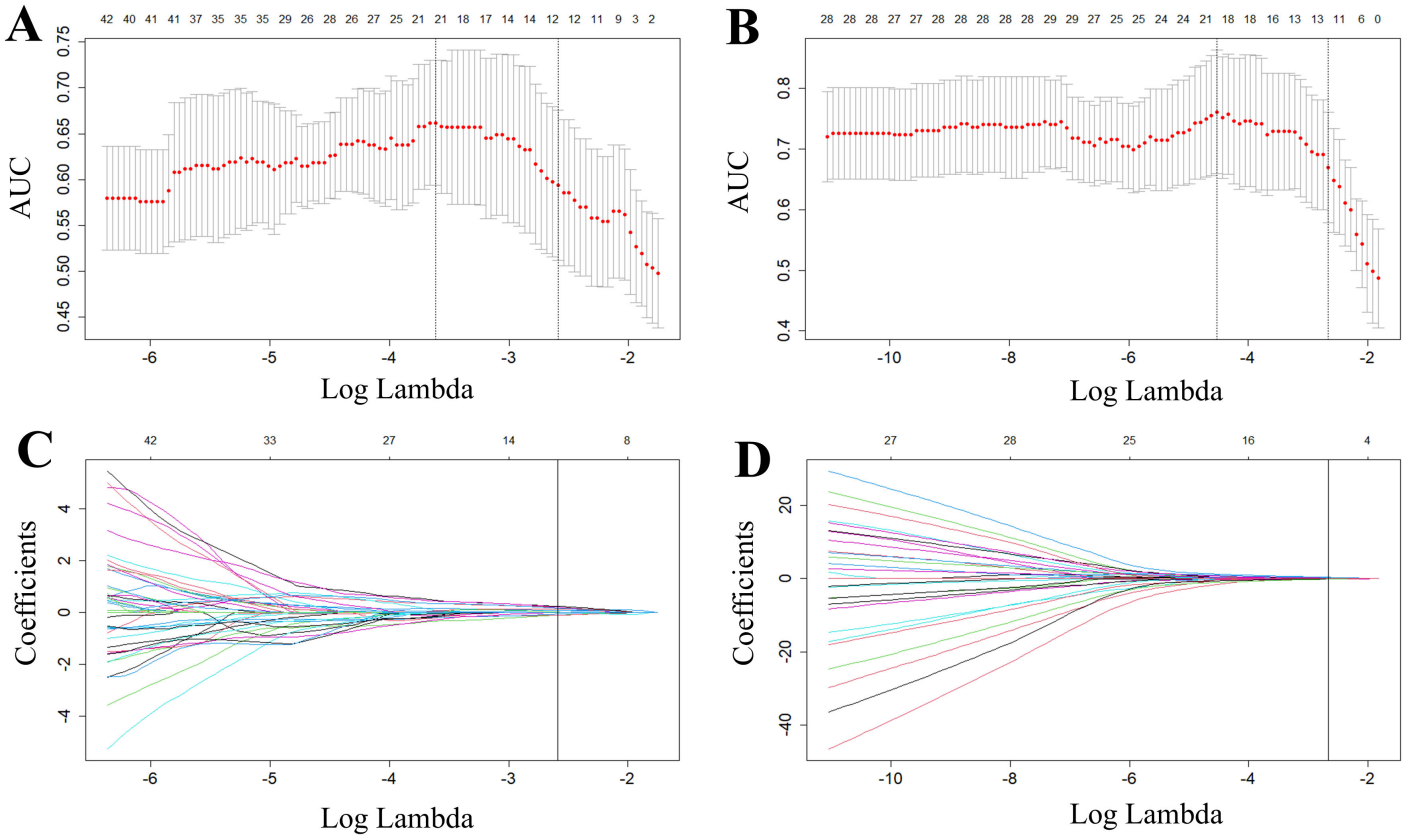
Radiomics features selected by LASSO regression. **(A, B)** represent tuning parameter (lambda) selection in the LASSO used fivefold cross-validation in predicting HRD score **(A)** and HRD status **(B)**, respectively. **(C, D)** represent LASSO coefficient profiles of the features. The red dots in the **(A, B)** indicate the AUC values of the model at different lambda values. From left to right, the bottom horizontal coordinates of the two dashed lines correspond to the log(lambda) values when the mean square error is minimized and when the standard error is doubled, respectively. Each curve in **(C, D)** represents one of the input features that was retained after the Mann-Whitney *U* test. The top horizontal coordinates of all four panels represent the number of retained features.

**Figure 4:**
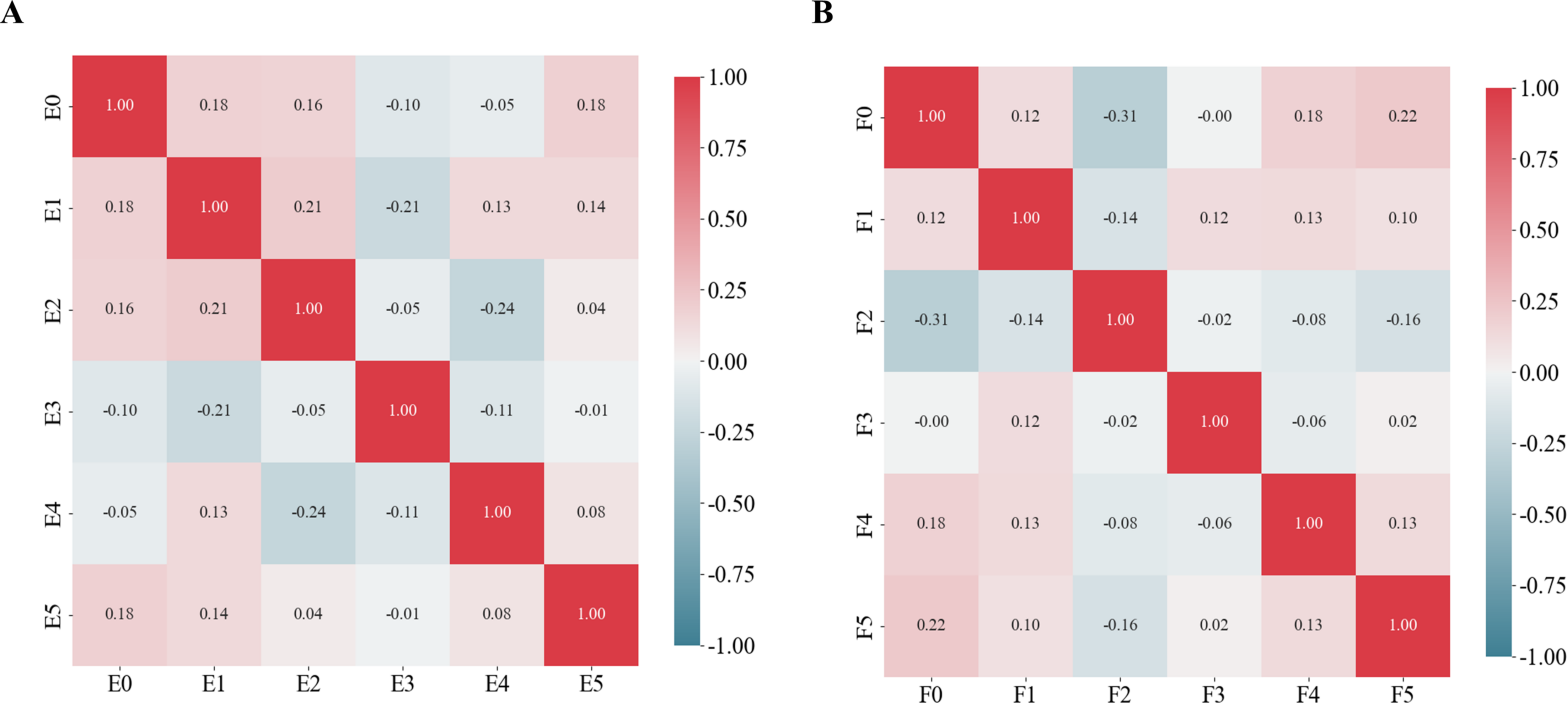
Pearson correlation coefficients for the selected features. **(A)** Schematic representation of the radiomics feature Pearson correlation coefficients chosen for predicting HRD scores. **(B)** Schematic representation of the radiomics feature Pearson correlation coefficients chosen for predicting HRD status.

For predictions of the HRD score and status, 12 and 13 features were retained after ICC, Mann-Whitney *U*-test and LASSO regression, respectively. **Figure 3** shows the feature selection using LASSO regression. The retained 12 and 13 features were further filtered using stepwise regression with AIC as the stopping rule, and finally 6 (for prediction of HRD score) and 6 features (for prediction of HRD status) were retained as the best subset of features and fitted to the final logistic regression model, respectively. For prediction of HRD score, 4 and 2 features were selected as the most important predictors from the intra- and peritumoral regions, respectively. For prediction of HRD status, 4 and 2 features were selected from the intra- and peritumoral regions, respectively. **Table 2** showed performance of all selected features. **Figure 4** demonstrates the Pearson correlation coefficients for all features; the correlation coefficients between two by two for all manual features, both for predicting HRD status and predicting HRD scores, are less than 0.6, which indicates that there is no high correlation between the features.

**Table 2:** Performances of the selected features for predicting HRD score and HRD status.

Feature	Region	Cohort	Mean ± SD		Mean ± SD		AUC	*P*
			High HRD score	Low HRD score	HRD-positive	HRD-negative		
log-sigma-1-0-mm-3D_ngtdm_Contrast (E0)	Intra	Training	0.92 ± 0.02	0.08 ± 0.03	–	–	0.679	0.010
		Validation	0.09 ± 0.03	0.10 ± 0.11	–	–	0.517	0.878
log-sigma-3-0-mm-3D_glszm_LowGrayLevelZoneEmphasis (E1)	Intra	Training	0.33 ± 0.12	0.26 ± 0.10	–	–	0.649	0.033
		Validation	0.26 ± 0.12	0.34 ± 0.09	–	–	0.769	0.006
Wavelet-LHH_glszm_LowGrayLevelZoneEmphasis (E2)	Intra	Training	0.56 ± 0.16	0.48 ± 0.14	–	–	0.678	0.011
		Validation	0.44 ± 0.18	0.59 ± 0.09	–	–	0.701	0.046
square_glcm_JointAverage (E3)	Intra	Training	1.94 ± 0.54	2.36 ± 0.73	–	–	0.675	0.012
		Validation	2.01 ± 0.37	1.74 ± 0.55	–	–	0.677	0.081
square_glrlm_ShortRunLowGrayLevelEmphasis (E4)	Peri	Training	0.18 ± 0.11	0.11 ± 0.08	–	–	0.684	0.008
		Validation	0.12 ± 0.64	0.12 ± 0.07	–	–	0.566	0.528
Wavelet-LLL_gldm_ DependenceVariance (E5)	Peri	Training	32.77 ± 8.68	27.56 ± 10.65	–	–	0.682	0.009
		Validation	30.19 ± 5.11	34.39 ± 8.27	–	–	0.653	0.135
square_glszm_GrayLevelNonUniformityNormalized (F0)	Intra	Training	–	–	0.70 ± 0.20	0.91 ± 0.16	0.671	0.019
		Validation	–	–	0.83 ± 0.17	0.59 ± 0.15	0.800	0.007
wavelet-HHH_glszm_GrayLevelNonUniformity (F1)	Intra	Training	–	–	7.67 ± 3.78	10.05 ± 4.21	0.663	0.025
		Validation	–	–	7.09 ± 4.57	10.81 ± 5.35	0.719	0.053
original_glszm_SmallAreaEmphasis (F2)	Intra	Training	–	–	0.39 ± 0.13	0.44 ± 0.11	0.662	0.026
		Validation	–	–	0.41 ± 0.07	0.43 ± 0.14	0.752	0.025
wavelet-LLL_glcm_Imc2 (F3)	Intra	Training	–	–	0.49 ± 0.10	0.38 ± 0.16	0.690	0.009
		Validation	–	–	0.47 ± 0.10	0.48 ± 0.12	0.490	0.950
square_glszm_SizeZoneNonUniformityNormalized (F4)	Peri	Training	–	–	0.16 ± 0.20	0.14 ± 0.06	0.640	0.007
		Validation	–	–	0.14 ± 0.04	0.15 ± 0.04	0.560	0.983
exponential_glszm_GrayLevelNonUniformityNormalized (F5)	Peri	Training	–	–	0.50 ± 0.35	1.00 ± 0.32	0.697	0.041
		Validation	–	–	0.65 ± 0.34	0.73 ± 0.35	0.495	0.603

SD, standard deviation; Intra, intratumoral; Peri, peritumoral.

### 3.3 Development of the radiomics signature

RSs were derived from intra- and peritumoral regions separately and in combination. Relevant features with non-zero coefficients in the logistic regression model were selected based on the training cohort. The formulas for predicting HRD score was calculated as follows:

Intra-RS-Score=-30.12 + 6.72E-4×original_ngtdm_Busyness+17.15×wavelet-LLH_glszm_SmallAreaEmphasis -909.70×wavelet-HLH_glrlm_GrayLevelVariance+989.86 ×wavelet-HHL_glcm_JointEnergy-66.66×log-sigma-5-0-mm-3D_gldm_SmallDependence

-HighGrayLevelEmphasis-1.03E-04×original_glrlm_LongRunHighGrayLevelEmphasis

+ 6.64E-01×original_glszm_ZoneEntropy

Peri-RS-Score=7.96 + 2.01×lbp-2D_firstorder_10Percentile+9.76×square_glrlm_

ShortRunLowGrayLevelEmphasis+0.41×gradient_glszm_SmallAreaHighGrayLevelEmphasis -26.03×wavelet-HHH_glszm_GrayLevelNonUniformityNormalized

Com-RS-Score=-6.53 + 25.98×log-sigma-1-0-mm-3D_ngtdm_Contrast+4.6×1log-sigma-3-0-mm-3D_glszm_LowGrayLevelZoneEmphasis+5.05×wavelet-LHH_glszm_LowGrayLevelZone-

Emphasis+6.36×square_glrm_ShortRunLowGrayLevel Emphasis +0.05×Wavelet-LLL_gldm_DependenceVariance-0.79×square_glcm-JointAverage.

The formulas for predicting HRD status were calculated as follows:

Intra-RS- Status= -353.04 + 1141.31×wavelet-HHH_firstorder_Mean+ 555.07×wavelet-HHL_gldm_LowGrayLevelEmphasis+5.93×wavelet-LHH_glszm_LowGrayLevelZoneEmphasis+6.21wavelet-LLL_glcm_MCC

Peri–RS-Status=3.17E-01-14.26×log-sigma-3-0-mm-3D_glcm_InverseVariance-9.71 ×wavelet-HHH_glszm_SmallAreaEmphasis+0.08×logarithm_firstorder_Skewness +2.91 ×wavelet-HHL_firstorder_Skewness+6.67×log-sigma-5-0-mm-3D_glcm_MaximumProbability

Com-RS-Status=5.22 + 10.47×square_glszm_SizeZoneNonUniformityNormalized-0.21×wavelet-HHH_glszm_GrayLevelNonUniformity+4.24×wavelet-LLL_glcm_Imc2-3.66×square_glszm_SizeZoneNonUniformityNormalized-2.54×exponential_glszm_

GrayLevelNon Uniformity Normalized-5.32×original_glszm_SmallAreaEmphasis.

### 3.4 Comparisons of intra- and peritumoral regions and their combination

**Figure 5:**
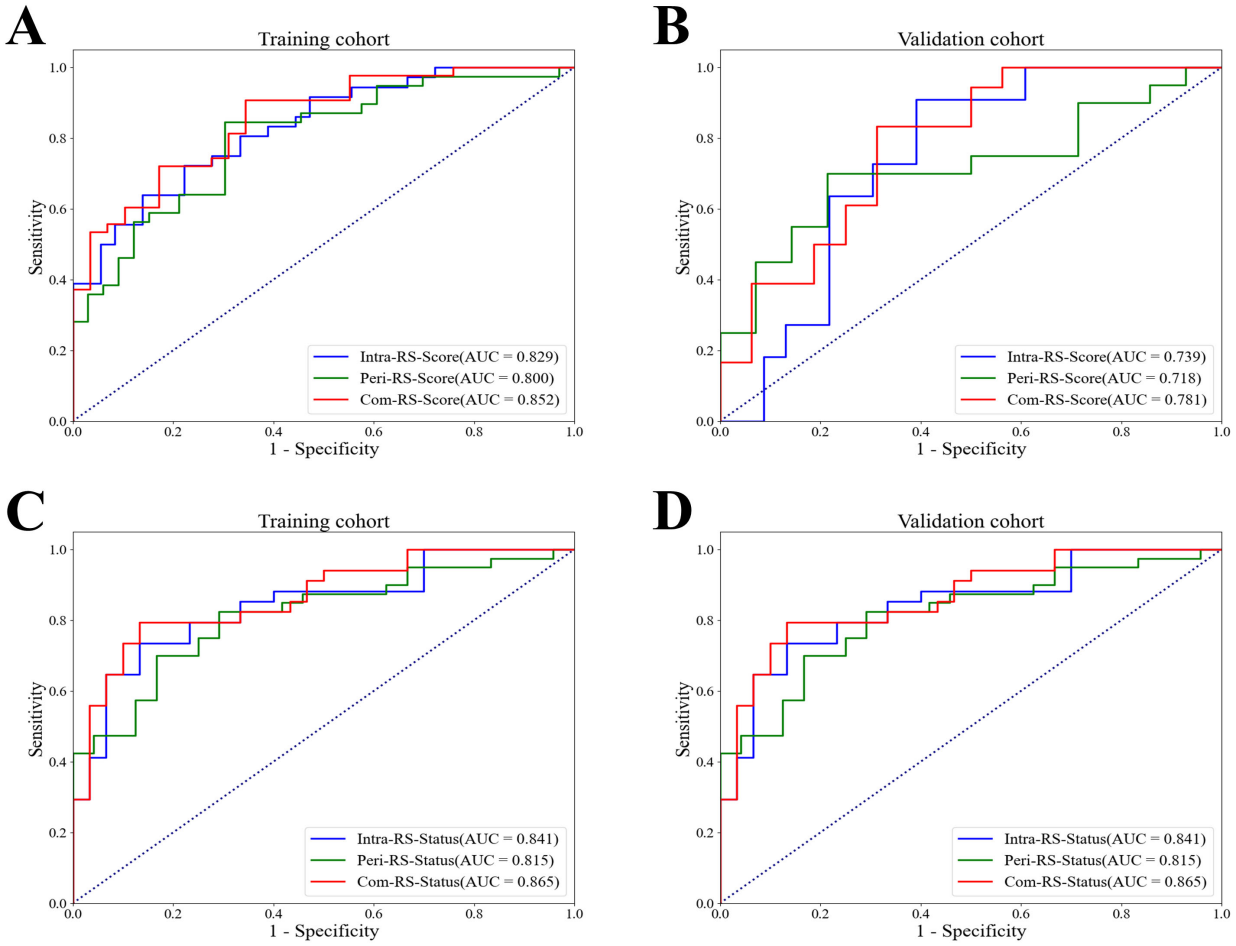
ROC curves of the Intra-RS-Score, Peri-RS-Score and Com-RS-Score for predicting the HRD score and HRD status. **(A, B)** represent ROC curves of the Intra-RS-Score (blue line), Peri-RS-Score (green line) and Com-RS-Score (red line) for predicting the HRD score; **(C, D)** represent ROC curves of the Intra-RS-Status (blue line), Peri-RS-Status (green line) and Com-RS-Status (red line) for predicting the HRD status; **(A, C)** corresponds to the training cohort, whereas the **(B, D)** corresponds to the validation cohort.

**Figure 6:**
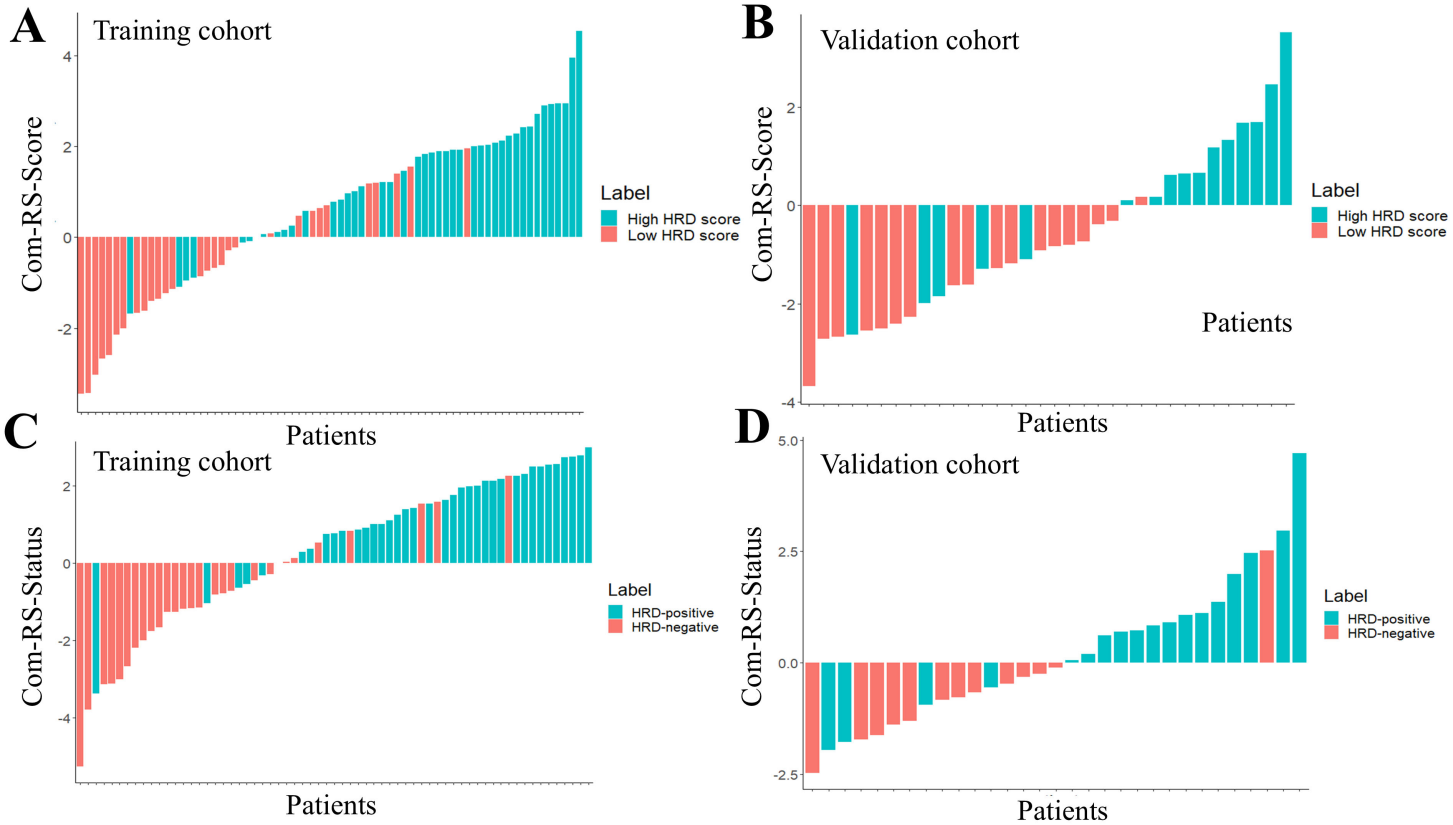
The waterfall plots for predicting HRD score and HRD status. The Com-RS-Score and Com-RS-Status of each patient are represented by colored bars, **(A, B)** represent the waterfall plots of the training and validation sets, respectively, for predicting HRD scores, and **(C, D)** represent the waterfall plots of the training and validation sets, respectively, for predicting HRD status.

**Table 3** showed comparisons of overall prediction performance of each RS. For predicting the HRD score, the Intra-RS-Score showed higher prediction abilities than Peri-RS-Score in terms of AUCs. The Com-RS-Score incorporating features from both intra- and peritumoral regions can significantly improve predictive AUCs compared with the Intra-RS-Score or Peri-RS-Score alone. It is worth noting that the Com-RS-Score has an overall higher SEN than the Intra-RS-Score and the Peri-RS-Score. However, in the training cohort, the increase in SEN comes at the cost of a significant decrease in SPE, and thus the ACC is only slightly improved, suggesting that the Com-RS-Score is identifying positive cases is more sensitive, but also leads to an increase in false positive cases. Similar trends were also observed in the validation cohort. To address this issue, future research should focus on optimizing radiomics feature selection and integration, and using more data or refined extraction methods to enhance positive case identification while minimizing false positives. Meanwhile, Intra-RS-Status was more predictive than Peri-RS-Status for predicting HRD status, and Com-RS-Status combining intra- and peritumoral regional features significantly improved the prediction of AUC compared to Intra-RS-Status or Peri-RS-Status alone. Additionally, the SEN and SPE of all three models were relatively balanced. **Figure 5** showed ROC curves of each RS. To demonstrate the value of our models, we plotted waterfall diagrams based on Com-RS. As shown in the **Figure 6**, most patients’ HRD score and status can be correctly distinguished by our RSs.

**Table 3:** Performances of the Intra-RSs, Peri-RSs and Com-RSs for predicting HRD score and HRD status.

	Training cohort				Validation cohort			
Model	AUC (95% CI)	ACC	SEN	SPE	AUC (95% CI)	ACC	SEN	SPE
HRD score								
Intra-RS-Score	0.829 (0.738-0.921)	0.750	0.722	0.778	0.739 (0.571-0.907)	0.716	0.609	0.909
Peri-RS-Score	0.800 (0.697-0.902)	0.750	0.846	0.697	0.718 (0.542-0.894)	0.676	0.700	0.786
Com-RS-Score	0.852 (0.765-0.938)	0.736	0.907	0.655	0.781 (0.621-0.941)	0.716	0.688	0.833
HRD status								
Intra-RS-Status	0.841 (0.744-0.939)	0.766	0.735	0.867	0.786 (0.615-0.956)	0.774	0.762	0.800
Peri-RS-Status	0.815 (0.710-0.919)	0.750	0.825	0.708	0.758 (0.586-0.930)	0.613	0.733	0.750
Com-RS-Status	0.865 (0.776-0.953)	0.797	0.794	0.867	0.800 (0.637-0.963)	0.710	0.714	0.800

AUC, area under the receiver operating characteristic curve; CI, confidence interval; ACC, accuracy; SEN, sensitivity; SPE, specificity; Intra-RS-Score, intratumoral-radiomics signature for predicting scores; Peri-RS, peritumoral-radiomics signature for predicting scores; Com-RS-Score, combined- radiomics signature for predicting scores; Intra-RS-Status, intratumoral-radiomics signature for predicting status; Peri-RS-Status, peritumoral-radiomics signature for predicting status; Com-RS- Status, combined- radiomics signature for predicting status; *, *P*<0.05.

### 3.5 Construction and evaluation of radiomics nomogram

**Figure 7:**
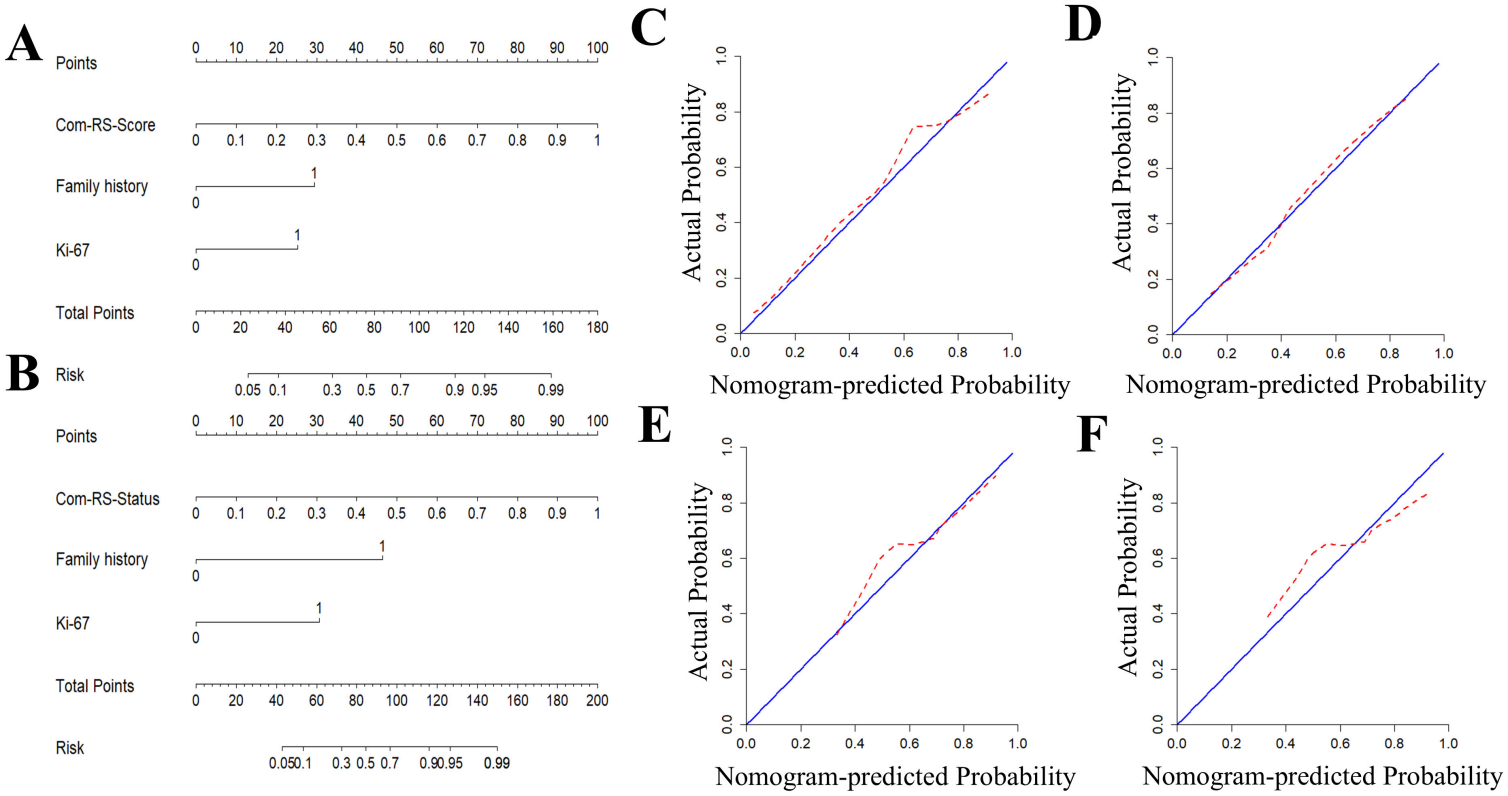
Development and validation of radiomics nomogram and Calibration curves for predicting HRD score and HRD status. **(A, B)** are column line plots for predicting HRD score **(A)** and HRD status **(B)**, respectively. Calibration curves for predicting HRD score and HRD status. **(C, D)** are calibration curves for predicting HRD score’s column line plots in the training **(C)** and validation cohorts **(D)**. **(E, F)** are calibration curves for predicting HRD status’column line plots in the training **(E)** and validation cohorts **(F)**.

Radiomics nomograms (**Figure 7**) were formulated based on Com-RS-Score and Com-RS-Status, family history and Ki-67, aiming to facilitate clinical practitioners in using our radiomics models to predict the HRD score (**Figure 7A**) and HRD status (**Figure 7B**). For predicting the HRD score, the second row represents Com-RS-score, and rows third to fourth represent family history and Ki-67, respectively. The risk of high-HRD score (**Figure 7A**) for each patient can be derived by combining Com-RS, family history and Ki-67 in the last row of the nomogram. For predicting the HRD status, we integrated Com-RS, family history with Ki-67 for constructing the nomogram.

**Figure 8:**
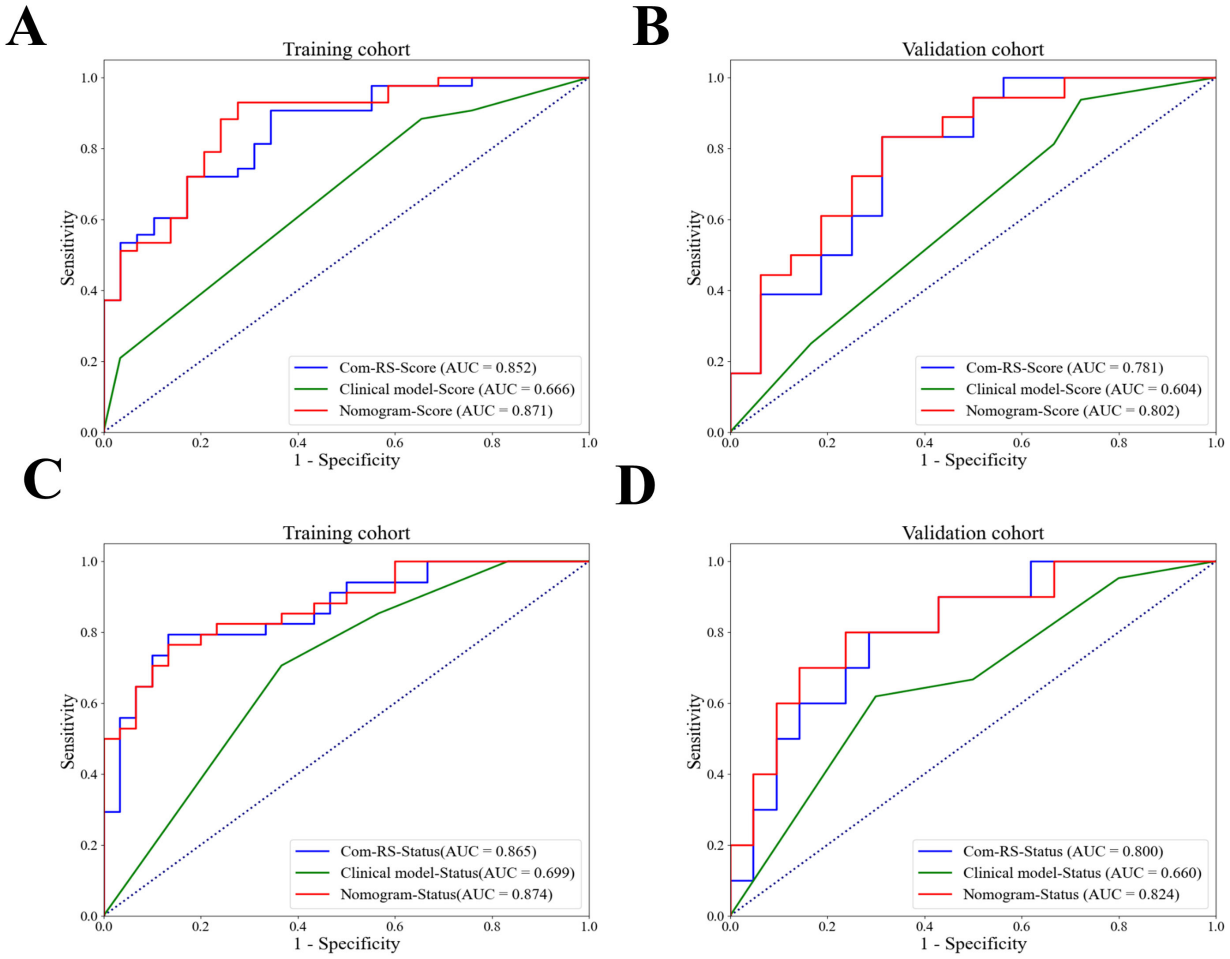
ROC curves for Com-RS-Score, Clinical model-Score and Nomogram-Score. ROC curves for Com-RS-Score, Clinical model-Score and Nomogram-Score in the training **(A)** and validation **(B)** cohort, and ROC curves for Com-RS-Status, Clinical model-Status and Nomogram-Status in the training **(C)** and validation **(D)** cohort.

**Figure 9:**
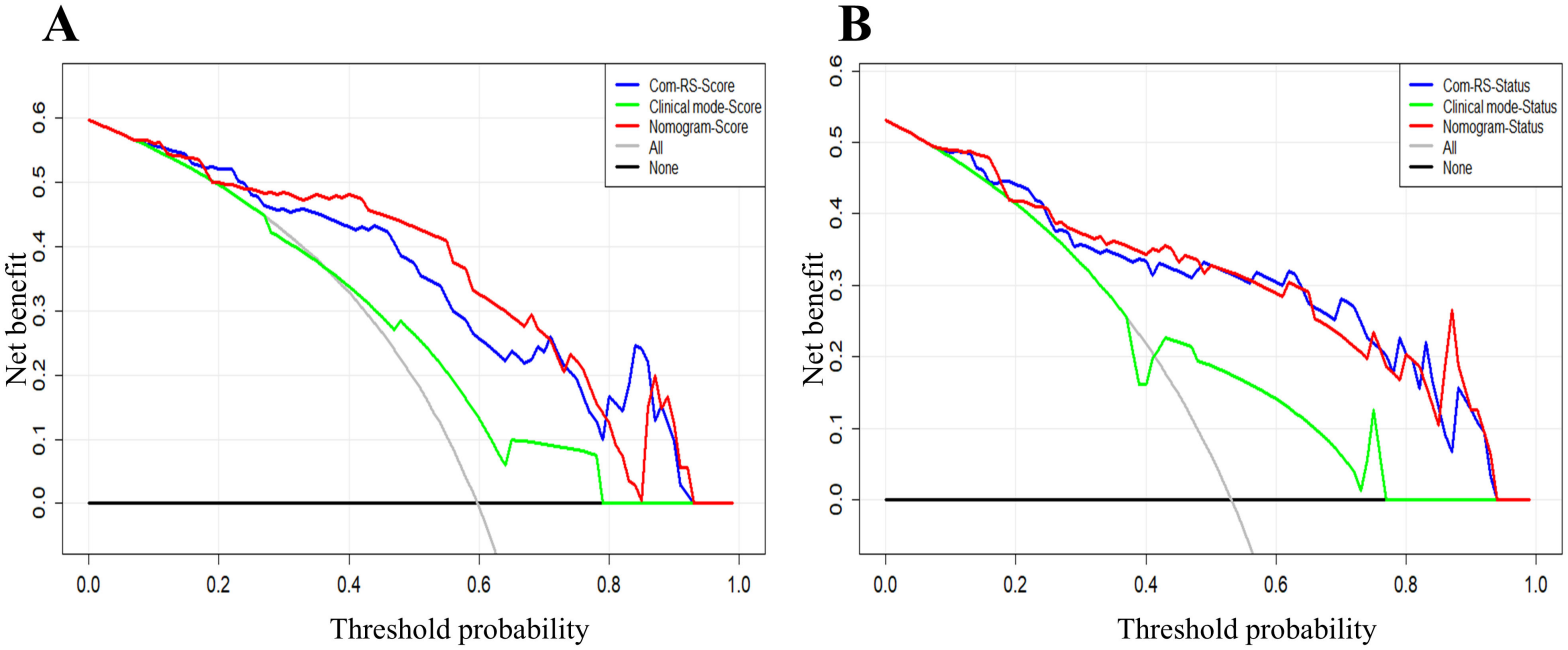
DCA curves for the developed Com-RSs, clinical models and nomograms. **(A)** HRD score prediction. **(B)** HRD status prediction. The x-axis represents the threshold probability, whereas the y-axis measures the net benefit. The black line represents the hypothesis that all patients were low HRD score **(A)** and HRD negative **(B)**. The gray line indicates the assumption that all patients were high HRD score **(A)** and HRD positive **(B)**.

**Table 4** compares prediction performances of Com-RSs, clinical models, and nomograms. For predicting HRD score, the nomogram had the best predictive performance, followed in order by Com-RS-Score and clinical model in both training (nomogram vs. Com-RS-Score vs. clinical model, 0.871vs.0.852 vs.0.666) and validation cohorts (nomogram vs. Com-RS-Score vs. clinical model, 0.802vs.0.781 vs.0.604). For predicting HRD status, the nomogram had the best predictive performance, followed in order by Com-RS and clinical model in both training (nomogram vs. Com-RS-Status vs. clinical model, 0.874 vs. 0.865 vs. 0.699) and validation cohorts (nomogram vs. Com-RS-Status vs. clinical model, 0.824 vs. 0.800 vs. 0.660). Additionally, compared to the clinical model, the Nomogram shows significantly higher ACC, SEN, and SPE in predicting both HRD status and HRD score. These results suggest that radiomics, which captures subtle image features not visible to the naked eye, and the inclusion of these features help overcome the limitations of traditional clinical models, thereby enhancing predictive performance. Calibration curves demonstrated good agreements between the nomogram-predicted and actual values in predicting HRD score (**Figures 7C, D**) and HRD status (**Figures 7E, F**). ROC curves in **Figure 8** provide an intuitive comparison of the predictive performance of each model. Finally, DCA curves in **Figure 9A** showed that the nomogram had a better net benefit in predicting HRD status compared to Com-RS-score and the clinical model when the threshold probability range of 0.30–0.70. Meanwhile, DCA curves in **Figure 9B** showed that the nomogram had a better net benefit in predicting HRD status when the threshold probability range of 0.25-0.45.

**Table 4:** Comparisons of the Com-RSs, clinical models and nomograms for predicting HRD score and HRD status.

	Training set				Validation set			
Model	AUC (95% CI)	ACC	SEN	SPE	AUC (95% CI)	ACC	SEN	SPE
M1	0.852 (0.765-0.938)	0.736	0.907	0.655	0.781 (0.621-0.941)	0.716	0.688	0.833
M2	0.666 (0.561-0.770)	0.667	0.655	0.884	0.604 (0.427-0.780)	0.559	0.278	0.938
M3	0.871 (0.788-0.954)	0.833	0.724	0.930	0.802 (0.651-0.953)	0.735	0.833	0.668
M4	0.865 (0.776-0.953)	0.797	0.794	0.867	0.800 (0.637-0.963)	0.710	0.714	0.800
M5	0.699 (0.633-0.769)	0.672	0.706	0.633	0.660 (0.461-0.859)	0.645	0.619	0.700
M6	0.874 (0.790-0.957)	0.797	0.765	0.867	0.824 (0.663-0.985)	0.774	0.762	0.800

AUC, area under the receiver operating characteristic curve; CI, confidence interval; ACC, accuracy; SEN, sensitivity; SPE, specificity; M1, Com-RSs-score; M2, clinical model for HRD score; M3, nomogram for HRD score; M4, Com-RSs-status; M5, clinical model for HRD status; M6, nomogram for HRD status; *, *P*<0.05.

## 4 Discussion

This study built and validated radiomics models based on the CT image for noninvasive prediction of HRD score and HRD status. In contrast to previous works that were focusing solely on the intra tumoral region of OC (23, 24, 26), our study analyzed radiomics features from both intra− and peritumoral areas and fusion to build radiomics models, with the aim of exploring whether the region surrounding OC contains potential information correlated to HRD. Results showed that the AUC of Com-RS-Score was higher than that of Intra-RS-Score and Peri-RS-Score for predicting HRD score; and the AUC of Com-RS-Status was higher than that of Intra-RS-Status and Peri-RS-Status for predicting of HRD status, which suggests that intra− and peritumoral regions can provide complementary information associated HRD score and status. The developed models may be helpful to guide the physicians in developing individualized maintenance therapy regimens for OC patients (36–38).

We comprehensively analyzed 1688 radiomics features from intra− and peritumoral regions of OC. This far exceeds previous studies that analyzed only 217 features (26) and 696 features from intratumoral regions in CT (39). For predicting HRD score, we identified 4 and 2 most important features from intra- and peritumoral regions, respectively, all belong to the textural feature class, which are based on statistics and can provide great amount of details regarding the intratumoral heterogeneity (24). For predicting HRD status, we identified 4 and 2 textural features from the intra- and peritumoral, respectively. The features were all filtered features that cannot be understood by naked-eyes, which may explain why radiologists can hardly evaluate the HRD scores and status of OC through visual examinations on the CT image. The original_glszm_SmallAreaEmphasis is a measure of the distribution of small size zones, with a greater value indicative of more smaller size zones and more fine textures. Our results showed that the average value of this feature is higher in the HRD-negative group than that in the HRD-positive group, which may indicate that the CT images of HRD-positive ovarian tumors are not finely textured enough. For predicting HRD status, the Gray Level NonUniformity feature was calculated from intratumoral regions, which measures the similarity of gray-level intensity value, with a lower value indicating a greater similarity in intensity values within the tumor. Our results revealed that the average value of this feature was lower in the HRD-positive group than that in the HRD-negative group. This was partially in line with a recent effort on the prediction of *BRCA* gene mutations, which showed that *BRCA* gene mutation was associated with the gray value of the CT image of OC (40).

Clinical factors were analyzed separately for the two prediction tasks, and family history and Ki-67 were considered to be statistically different in predicting HRD score and HRD status. This was consistent with previous findings suggesting that the prevalence of *BRCA* gene mutations is highest in patients with a family history of OC, which may lead to HRD in patients (27). We found Ki-67 is a high-risk factor for high HRD score and HRD-positive (41). This is consistent with previous studies (42, 43), which suggest that Ki-67 may be a clinical factor associated with in patients with OC. The nomogram model, which integrates family history, Ki-67, and radiomics features, outperforms the Com-RS-Score in predicting HRD scores, with improved AUCs of 0.871 in the training and 0.802 in the validation cohort. In predicting HRD status, the nomogram also demonstrates superior performance compared to Com-RS-Status, with an AUC of 0.874 for the training and 0.824 for the validation cohort This suggests that the nomograms constructed in our study can help physicians in predicting HRD score and HRD status of OC.

There are limitations in this study. Firstly, this is retrospective research with samples from a single hospital. Validations with a larger sample size from multi centers would be conducted in our future work. We plan to implement a multi-center validation study with a larger and more diverse patient cohort. This will involve standardizing imaging protocols across participating centers to minimize variability in data collection and analysis. We will work closely with collaborating institutions to ensure consistency in imaging techniques, acquisition parameters, and post-processing methods, which are critical for the accurate and reliable assessment of our radiomic biomarkers. Secondly, although CT imaging is important in pelvic cavity screening, performance of other modalities for predicting HRD score and status should also be evaluated. Thirdly, manual segmentation of regions of interest is time-consuming and subjective, limiting research progress. To overcome this, future studies should integrate machine learning-based automated or semi-automated tumor segmentation methods, such as deep learning models like U-Net, transfer learning, and active learning. These strategies could improve accuracy, efficiency, and accelerate research. Finally, although we assessed intra- and peritumoral regions, the peritumoral region was generated with a dilation distance of 1 cm, and the establishment of a radiomics model based on the peritumoral region with different dilation distances could be a new direction for research.

In addition to these limitations, future research should focus on addressing how these findings can be effectively integrated into clinical workflows. While radiomics models have demonstrated promising performance, their real-world applicability in clinical settings will depend on overcoming challenges such as cost-effectiveness, infrastructure requirements, and staff training. For instance, substantial investment in advanced computing infrastructure and imaging equipment, as well as the development of user-friendly interfaces for clinical staff, is essential for the implementation of these models in clinical practice. Furthermore, training radiologists and oncologists to interpret radiomics data and integrate it with other clinical factors is crucial for successful adoption. By addressing these challenges, we can ensure that the models we have developed are reliably integrated into personalized treatment plans, ultimately improving outcomes for ovarian cancer patients and those with other cancers.

## 5 Conclusion

This study suggests that radiomics features of abdominal CT in OC can provide high discrimination efficiency for predicting patients’ HRD scores and HRD status. The proposed nomograms can help clinicians provide personalized treatment plans for patients.

## Data Availability

The datasets presented in this article are not readily available due to ethical restrictions involving patient privacy and hospital regulations. Requests to access the datasets should be directed to [Xingling,Wang, wangxingling@cancerhosp-ln-cmu.com].
